# Application opportunity of Doppler ultrasound combined with CT angiography in diabetic lower extremity arterial disease and the analysis of the risk factors

**DOI:** 10.3389/fendo.2023.1257241

**Published:** 2024-01-30

**Authors:** ShaoRui Zhang, Yan Wu, YuQing Guo, XinJu Jia, Yan Kang, XueLian Shen, Jia Song, AiGe Yang

**Affiliations:** ^1^ Department of Nutrition, The First Hospital of Zhangjiakou, Zhangjiakou, Hebei, China; ^2^ Department of Clinical Laboratory, The First Hospital of Hebei Medical University, Shijiazhuang, Hebei, China; ^3^ Department of Endocrinology, The First Hospital of Hebei Medical University, Shijiazhuang, Hebei, China

**Keywords:** type 2 diabetes, lower extremity arterial, angiography, Doppler ultrasound, application opportunity

## Abstract

**Objective:**

This study examined the potential of combining Doppler ultrasound (DUS) and CT angiography (CTA) for early detection and intervention of lower extremity arterial disease (LEAD) in diabetes.Concurrently, risk factors influencing LEAD progression were analyzed.

**Methods:**

106 Type-2 diabetes patients with LEAD, having undergone DUS and CTA, were divided into four stages according to Fontaine stage. Results of DUS and CTA were compared across stages and potential risk factors were analyzed.

**Results:**

Positive detection rates of LEAD differed between DUS and CTA for Fontaine stages I and II (P < 0.05), with no significant difference for stages III and IV (P > 0.05). CTA identified subgroups with mild to moderate stenosis and severe stenosis or occlusion, with positive rates on DUS of 17.95% and 89.9% respectively. Hypertension was found as an independent risk factor affecting LEAD progression.

**Conclusion:**

CTA should be performed early for LEAD in diabetes patients at Fontaine stages I and II, regardless of DUS results. For diabetes patients with LEAD, stringent blood pressure control is crucial to delay disease progression.

## 1 Introduction

Diabetes is a group of metabolic diseases characterized by chronic hyperglycemia caused by insulin deficiency and/or utilization disorders. Various complications such as kidney, eye, nerve, heart and lower extremity arterial will be caused by the metabolism disorder of carbohydrate, fat and protein (1). The hallmark of LEAD is the narrowing or obstruction of arteries supplying blood to the lower extremities, which can lead to reduced blood flow and, in severe cases, result in critical limb ischemia. Since only 10% - 20% of patients have typical performance, the awareness and treatment rate of LEAD are very low, which is an important cause of disability and death in diabetes (2). There is a study showed that the mortality rate of hospitalized patients with diabetes foot is as high as 14.4% (3), which not only seriously affects the quality of life of patients, but also greatly increases the economic burden on their families (4). So, early and accurate diagnosis of LEAD is crucial for timely intervention and improved patient outcomes. The choice of diagnostic modalities plays a pivotal role in identifying the disease, and among these, Doppler ultrasound (DUS) and CT angiography have emerged as valuable tools for assessing arterial pathology in diabetic individuals.

There are many methods to check LEAD. Among them, ABI, as the preferred screening method for LEAD in diabetes, is simple and inexpensive, and can be used to predict diabetic foot and cardiovascular and cerebrovascular diseases (5). However, due to the influence of vascular calcification, its sensitivity in the examination of LEAD in diabetes is not high (6). Although it has the advantages of non-invasive and convenient, the tortuous course and calcification of blood vessels as well as the level of operators will affect the results (7), which will easily lead to missed diagnosis. The combination of Doppler ultrasound and CT angiography offers an intriguing application opportunity in the realm of diabetic lower extremity arterial disease. These two diagnostic techniques provide complementary information, enabling a comprehensive evaluation of vascular health in diabetic patients. Doppler ultrasound excels in assessing blood flow, while CT angiography provides detailed anatomical information about the arterial system. Integrating these modalities allows for a more holistic understanding of the disease, aiding in its early detection and precise characterization. And CTA has less radiation than DSA, which has a high clinical value (8). However, due to high price, need for injection of contrast agents, and limitations of patients’ own conditions, CTA is not the preferred method. Therefore, it is absolutely important to choose appropriate timing of CTA examination in clinical practice. In this study, 106 patients with LEAD of diabetes in different Fontaine stages were examined with CTA and DUS, and we scrutinize the complex web of risk factors inherent to diabetes and how they interact with the diagnostic tools in use. Through this exploration, we aim to contribute valuable insights that can enhance the early detection and management of LEAD in diabetic individuals, ultimately improving patient care and outcomes in this challenging clinical landscape.

## 2 Materials and methods

### 2.1 Research object

A total of 106 type 2 diabetic LEAD patients from the First Hospital of Hebei Medical University from 2020 to 2022 were enrolled. According to the clinical symptoms of patients, 106 patients with LEAD of diabetes were divided into four stages according to Fontaine stage (8): General information and biochemical indicators were analyzed statistically based on patients. Imaging examinations were performed on each affected limb. This study has been reviewed and approved by the Ethics Committee of our hospital, and all research subjects have signed informed consent forms. The inclusion criteria and exclusion criteria were as follows:

#### 2.1.1 Inclusion criteria

1. Meet the diagnostic criteria of WHO type 2 diabetes in 1999 (9). 2. Symptoms or signs of decreased pulse of dorsalis pedis artery, numbness and cooling of lower limbs, fatigue of lower limbs, intermittent claudication, rest pain, ulceration or gangrene. 3. Lower extremity arteries DUS were performed after hospitalization, and CTA examination of lower extremity arteries was performed within one week. All patients had complete imaging data.

#### 2.1.2 Exclusion criteria

1. Can not cooperate to complete the examination. 2. With severe systemic diseases such as heart diseases, liver diseases, impaired kidney function (Serum Creatinine> 1.5 mg/dL or estimated glomerular filtration rate (eGFR)< 60 mL/min/1.73 m²), and blood diseases. 3. Allergic to iodine contrast agents used in CTA. 4. The LEAD caused by trauma. 5. Concurrent chronic or acute infections. 6. Breastfeeding women or pregnant women.

### 2.2 Research method

#### 2.2.1 General data

Patient data, such as gender, age, diabetes duration, smoking history, and comorbidities, were collected. Comorbidities comprised hyperlipidemia, hypertension, coronary heart disease (typified by angina pectoris, confirmed by coronary angiography or myocardial infarction history), and cerebrovascular disease (history of cerebral infarction or hemorrhage).

#### 2.2.2 Laboratory examination

Immune agglutination method (Siemens DCA2000+HbAlc analyzer) was used to measure glycosylated hemoglobin (HbAlc). The Beckman LX20 automatic biochemical analyzer was used to measure total cholesterol (TC), low-density lipoprotein cholesterol (LDL-C), triglycerides (TG), aspartate aminotransferase (AST), alanine aminotransferase (ALT), total bilirubin (TB), fasting blood glucose (FPG) Indicators such as creatinine (Cr) and urea nitrogen (BUN). The above tests are strictly carried out by professional personnel in accordance with the instructions.

#### 2.2.3 Measurement of DUS

The instrument adopted EPIQ7C color Doppler ultrasound diagnostic instrument produced by Philips. The patient took a comfortable position, observed the femoral artery, popliteal artery, anterior tibial artery, posterior tibial artery and dorsalis pedis artery, measured the inner diameter, intima media thickness (IMT), plaque, narrow and occlude. It mainly observes the filling of blood flow in the vascular cavity, and determine if there is any filling. When there is a defect or stenosis in the lumen, the local blood flow becomes irregular and thin, showing a colorful pattern. Spectral Doppler can detect a significant increase in blood flow velocity, and the blood flow signal is not full when occluded. The results underwent collaborative assessment by two seasoned ultrasound physicians. In instances where disparities or instances of inter and intraobserver variability emerged, a third ultrasound physician undertook the process of evaluation and validation.

#### 2.2.4 Measurement of CTA

The patient was placed in supine position and iodine contrast agent was injected through elbow vein. Name of contrast agent: Iodixanol Injection. Dosage: 30-60ml according to the patient’s age, weight, and physical condition. Side effects: The side effects associated with Iodixanol Injection are usually mild to moderate and transient. Severe reactions and deaths are only observed in very rare cases, including acute exacerbation of chronic kidney disease, acute kidney failure, anaphylactic shock or quasi anaphylactic shock, cardiac reactions after hypersensitivity reactions, and cardiac or cardiac respiratory arrest. During our study, no patients experienced the adverse reactions. The patient was scanned with SIEMENS SOMATOM Force dual source CT, and the scanned image was imported into the vascular image analysis software (itksnap, USA) immediately. The CTA diagnosis results are evaluated by two experienced radiologists, and when there are differences, the third radiologist explains them.

#### 2.2.5 Evaluation criteria

According to the degree of vascular stenosis, DUS and CTA were performed to divided patients with LEAD into normal, mild stenosis (stenosis rate<50%), moderate stenosis (stenosis rate 50% - 75%), severe stenosis (stenosis rate 76% - 99%), and occlusion (stenosis rate 100%). Stenosis rate determination: Detect a significant increase in blood flow velocity, record peak systolic flow velocity (PSV), and determine the presence and degree of stenosis. If the PSV is less than 150cm/s, it is judged as no stenosis. The most severe degree was taken when the degree of arterial stenosis in different segments was inconsistent.

### 2.3 Statistical methods

SPSS.27 was used for data analysis. The counting data was expressed in the number of cases or percentages, and the measuring data was expressed in Mean ± SD. Pairing χ2 was used to compare the difference between CTA and DUS, CTA and ABI respectively. Ordinal logistic regression analysis was used to analyze the risk factors of LEAD in diabetes. *P* < 0.05 was statistically significant.

## 3 Results

### 3.1 Patients’ characteristics

Among 106 type 2 diabetic LEAD patients, 77 were male and 29 were female. The age ranged from 35 to 90 (68.14 ± 9.76) years. There were 67 cases with hypertension (Systolic pressure≥140mmHg, diastolic pressure≥90mmHg), 21 cases with hyperlipidemia(Total cholesterol>6.2mmol/L), 30 cases with cerebrovascular disease and 28 cases with coronary heart disease determined by MRI and DSA. There were 22 cases in Fontaine I, 15 cases in Fontaine II, 34 cases in Fontaine III, and 35 cases in Fontaine IV (**Table 1**).

**Table 1:** Patients’ characteristics.

		Fontaine I	Fontaine II	Fontaine III	Fontaine IV	Total
Count		22	15	34	35	106
Gender	Male Female	17	12	23	25	77
		5	3	11	10	29
Age		66.20 ± 13.88	67.87 ± 6.76	69.50 ± 9.00	69.83 ± 7.48	68.14 ± 9.76
Diabetes duration(>10years)		14(63.64%)	10(66.67%)	22(64.71%)	27(77.14%)	73(68.87%)
Smoking history		11(50.00%)	8(53.33%)	11(32.35%)	10(28.57%)	40(37.74%)
Hypertension		8(36.36%)	8(53.55%)	22(64.71%)	29(82.86%)	67(63.21%)
Hyperlipidemia		6(27.27%)	4(26.67%)	7(20.59%)	4(11.43%)	21(19.81%)
Cerebral vascular disease		8(36.36%)	4(26.67%)	9(26.47%)	9(25.71%)	30(28.30%)
Coronary artery disease		2(9.09%)	6(40.00%)	11(32.35%)	9(25.71%)	28(26.42%)

Data are shown as Mean ± SD or n(%).

### 3.2 Comparison of DUS and CTA in patients with different fontaine stages

3.1 The positive detection results of CTA compared with DUS in patients with Fontaine I were significantly difference (40(91%) vs 22(50%), *P* < 0.001), and the positive detection results of CTA compared with DUS in patients with Fontaine II were significantly difference (28(93%) vs 17(57%), *P* < 0.003). In Fontaine III and IV patients, there was no significant difference between CTA and DUS in the positive detection results of LEAD in diabetes (*P* > 0.05) (**Table 2**).

**Table 2:** Comparison of detection rate of stenosis vessel for DUS and CTA in patients with different Fontaine stages.

	Total	CTA	DUS	χ2	*P*
Fontaine I	44	40	22	14.45	0.000*
Fontaine II	30	28	17	7.69	0.003*
Fontaine III	68	66	60	3.13	0.700
Fontaine IV	70	68	62	3.13	0.700

DUS, doppler ultrasound; CTA, CT angiography.

*DUS group compared with CTA group (P < 0.05).

**Figure 1:**
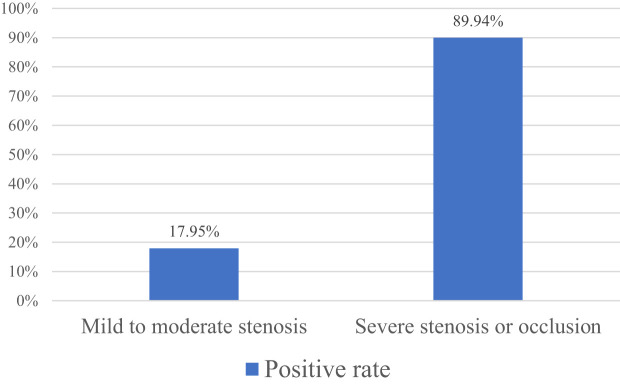
Detection rate of DUS on vessels with different degrees of stenosis.

3.2 The diseased vessels were divided into two subgroups: mild to moderate stenosis and severe stenosis or occlusion based on the results of CTA. Both subgroups were compared with the results of DUS. The results showed that the vessels CTA showed mild to moderate stenosis and the positive rate of stenosis on DUS was 17.95%; the vessels CTA showed severe stenosis or occlusion and the positive rate of stenosis on DUS was 89.94% (**Figure 1**).

### 3.3 Analysis of the risk factors that aggravate the progression of LEAD

Logistic regression analysis showed that hypertension was an independent risk factor affecting the progression of LEAD in diabetes (**Table 3**).

**Table 3:** Analysis of risk factors for aggravating LEAD in diabetes.

		Fontaine I	Fontaine II	Fontaine III	Fontaine IV	*P*
Age		66.20 ± 13.88	67.87 ± 6.76	69.50 ± 9.00	69.83 ± 7.48	0.424
Gender	Male	17(77.27%)	12(80.00%)	23(67.65%)	25(71.43%)	0.882
	Female	5(22.73%)	3(20.00%)	11(32.35%)	10(28.57%)	
Diabetes duration(>10years)		14(63.64%)	10(66.67%)	22(64.71%)	27(77.14%)	0.570
Smoking history		11(50.00%)	8(53.33%)	11(32.35%)	10(28.57%)	0.326
Hypertension		8(36.36%)	8(53.55%)	22(64.71%)	29(82.86%)	0.017*
Hyperlipidemia		6(27.27%)	4(26.67%)	7(20.59%)	4(11.43%)	0.466
Cerebral vascular disease		8(36.36%)	4(26.67%)	9(26.47%)	9(25.71%)	0.683
Coronary artery disease		2(9.09%)	6(40.00%)	11(32.35%)	9(25.71%)	0.912

Data are shown as Mean ± SD or n(%); LEAD, lower extremities arterial disease; *Comparison of four groups of the same indicator (P < 0.05).

### 3.4 The difference and correlation when used CTA and ultrasound scans examine LEAD

Color Doppler ultrasound (CDUS) examination shows that there is no color blood flow signal in the occluded segment lumen as a direct sign, and diffuse arterial lumen thickening, uneven intimal thickness, uneven surface, and a small amount of multicolored blood flow can be observed on distal Doppler flow imaging (CDFI); color doppler energy (CDE) shows single color and other signs, and small collateral circulation arteries can also be observed around the femoral popliteal artery in chronic arterial occlusion, with slender colored blood columns entering the distal lumen of the occlusion; pulsed wave doppler (PW) imaging shows that the arterial spectrum cannot be detected in the occluded segment lumen, the low-speed unidirectional single peak spectrum is present in the distal end lumen, and most of the unidirectional peak waves are present in the proximal end lumen. Multi slice spiral CTA display: Continuous interruption of blood vessels in the affected segment of the lower limb artery, with no contrast enhancement in the corresponding area; Simultaneously display the course and origin of the collateral arteries. CTA, especially in arterial wall calcification or peripheral veins When venous stones are formed, these abnormal calcification shadows can be clearly seen, but there is a certain amount of ionizing radiation present. At all, CDUS can accurately display stenosis and occlusion without significant difference from angiography, due to the comparative study of CDUS, CTA, and DSA in the diagnosis of arterial diseases around the vessel wall and in the lower limbs, CTA is superior to other imaging methods in accurate measurement of longer lesions and display of collateral circulation arterioles.

### 3.5 The limitations and constraints encountered when performing CTA and ultrasound scans.

DUS cannot provide detailed information about soft tissues and organs like magnetic resonance imaging and CT. During the examination process, some patients may experience poor image reliability due to obesity or other factors, which may affect the accurate diagnosis of the lesion. Therefore, multiple examination methods need to be combined to comprehensively determine the condition. The CTA have some contraindications as follows: Iodine allergy, hyperthyroidism and asthma patients; Severe heart, liver, and kidney dysfunction.

## 4 Discussion

The incidence rate of diabetes is rapidly increasing. It is predicted that by 2045, there will be over 700 million people worldwide suffering from diabetes (10). Lower extremity arterial disease (LEAD), a complication of diabetes with no obvious clinical symptom in the early stage (11), is a significant public health concern. Digital subtraction angiography (DSA) is the gold standard for diagnosing LEAD clinically, but because it’s invasive and could potentially cause vascular injuries and other complications, this examination method can be difficult for many patients to accept (12). In comparison, Computed Tomography Angiography (CTA) and Doppler ultrasound (DUS) are non-invasive examination methods. CTA has a high sensitivity and specificity in diagnosing LEAD, with a sensitivity and specificity of 95% and 96% respectively for blood vessels with stenosis greater than 50% (13). However, the high cost and nephrotoxicity of the contrast agent used in CTA limit its widespread use in clinical practice (14). While the accuracy of DUS in detecting narrowed blood vessels is not as high as CTA, it still remains the preferred examination method for diabetic LEAD due to its low cost, non-radiation, and non-toxicity.In this study, there were more patients in the Fontaine III and Fontaine IV groups, which with more severe symptoms. This is consistent with the phenomenon in clinical practice. The patients do not pay attention to the symptoms when they are mild. They will not go to see a doctor until the disease develops to a later stage, at which the prognosis of the disease is often poor. In addition, we found that some patients already have severe vascular stenosis when only the symptoms are mild, which may be related to the sensory retardation of patients with diabetic peripheral neuropathy. Previous study has suggested that about 73.7% of diabetic foot patients are also complicated with diabetic peripheral neuropathy (3), which makes the patients’ limbs hypoesthesia and reduce the attention to the disease, thus leading to the further progress of LEAD.

In previous studies, DSA was used as the gold standard to compare the diagnostic accuracy of DUS and CTA in LEAD. The results showed that CTA was more sensitive, specific and accurate than DUS (15, 16). Rahul Dev et al. revealed that in patients suspected of PAD, the results of CTA and DUS were different (17). However, no previous research has shown that the difference between the two is throughout the whole process of LEAD in diabetes or just exists in a certain stage of the disease. According to the Fontaine stage, the patients with LEAD of diabetes included in this study were divided into Fontaine I - IV stages. After statistical analysis, it was found that in patients with different Fontaine stages, the positive detection results of CTA and DUS for stenotic vessels in patients with diabetic LEAD in stages I and II were less consistent, while the results of stages III and IV were more consistent, which indicated that in patients in stages I and II whose symptoms are lighter, the sensitivity of DUS is lower than that of CTA. As the symptoms of the patient worsen and the degree of vascular stenosis increases, the consistency between the two methods in detecting narrow blood vessels becomes higher. To the further analysis of the reasons for the differences between the two methods, the diseased vessels were divided into two subgroups: mild to moderate stenosis and severe stenosis or occlusion based on the results of CTA. Both subgroups were compared with the results of DUS. The results showed that the vessels CTA showed mild to moderate stenosis and the positive rate of stenosis on DUS was 17.95%; the vessels CTA showed severe stenosis or occlusion and the positive rate of stenosis on DUS was 89.9%. And the number of stenotic artery segments detected by CTA was far more than that by DUS, indicating that DUS is less sensitive to mild and moderate stenosis, and more sensitive to severe stenosis. CTA is superior to DUS in detecting stenotic vessels of diabetic LEAD. At the same time, this study observed that a few patients with severe symptoms and already confirmed by CTA that exist artery stenosis were not detected by DUS. If these patients were not further examined, but were mistaken for lower limb pain caused by lumbar spinal stenosis, osteoarthritic pain, osteoporosis or other diseases, which would delay treatment, causing disease progression and deterioration. Therefore, in patients with existing clinical symptoms but no indication of arterial stenosis on DUS, especially those in Fontaine I and II, further CTA examination should be actively performed.

Previous studies have agreed that age, male, course of diabetes, smoking history and hypertension are independent risk factors for diabetic LEAD (18, 19). Lavdim H. Ymeri discovered that hypertension was associated with threefold increased risk for arterial stenosis of the lower extremities (11). Another study found that about 1/4 of 199 patients with acute ischemic stroke were diagnosed as LEAD (20). The risk factors that promote the incidence of LEAD in diabetes have been studied in the past, but no research has been conducted to explore the risk factors that aggravate the disease at present. In this study, we found that although age, male, smoking history, course of diabetes, and cerebrovascular disease were important factors of diabetic LEAD, they had little relationship with the progress of LEAD. Only hypertension was the independent factor that aggravated the development of LEAD. Therefore, patients with diabetic LEAD should strictly control their blood pressure to delay the further progress of the disease.

Limitations: The number of cases in this study is small, and the study samples are inpatients. There may be some errors in the research results. The number of cases needs to be increased in the future to further explore the application of CTA combine with DUS in diabetic LEAD.

## 5 Conclusions

1. For patients with LEAD of diabetes in Fontaine stage I and II, even if DUS shows no obvious vascular stenosis, CTA examination of lower extremity artery should be performed at an early stage for early prevention, diagnosis and treatment.

2. Diabetes patients with LEAD should strictly control blood pressure to delay the progress of the disease.

## Data availability statement

The original contributions presented in the study are included in the article/supplementary material. Further inquiries can be directed to the corresponding author.

## Ethics statement

The studies involving humans were approved by ethics committee of The First Hospital of Hebei Medical University. The studies were conducted in accordance with the local legislation and institutional requirements. The participants provided their written informed consent to participate in this study.

## Author contributions

SZ: Conceptualization, Data curation, Formal analysis, Investigation, Writing – original draft. YW: Conceptualization, Data curation, Formal analysis, Methodology, Resources, Writing – original draft. YG: Data curation, Formal analysis, Validation, Writing – review & editing. XJ: Resources, Supervision, Visualization, Writing – original draft. YK: Formal analysis, Investigation, Project administration, Writing – review & editing. XS: Data curation, Formal analysis, Methodology, Writing – review & editing. JS: Data curation, Formal analysis, Validation, Writing – original draft. AY: Conceptualization, Methodology, Project administration, Writing – review & editing.
